# The concept of natural genome reconstruction.Part 6. Analysis of changes in the frequency of occurrence of SNPs in exons of the genes associated with clonal hematopoiesis of uncertain potential in a patient with a neuroendocrine tumor of the small intestine in the terminal stage of progression after modification of the hematopoietic stem cell genome with hDNAgr (a clinical case report)

**DOI:** 10.18699/vjgb-26-57

**Published:** 2026-07

**Authors:** D.Y. Oshchepkov, V.S. Ruzanova, S.G. Oshikhmina, A.A. Traspov, E.V. Dolgova, G.S. Ritter, S.S. Kirikovich, E.V. Levites, Y.R. Efremov, O.S. Taranov, L.U. Grivtsova, S.V. Sidorov, S.D. Nikonov, O.Y. Leplina, A.A. Ostanin, E.R. Chernykh, N.A. Kolchanov, A.S. Bryukhovetskiy, S.S. Bogachev

**Affiliations:** Institute of Cytology and Genetics of the Siberian Branch of the Russian Academy of Sciences, Novosibirsk, Russia; Institute of Cytology and Genetics of the Siberian Branch of the Russian Academy of Sciences, Novosibirsk, Russia; Institute of Cytology and Genetics of the Siberian Branch of the Russian Academy of Sciences, Novosibirsk, Russia Novosibirsk State University, Novosibirsk, Russia; National Medical Research Radiological Centre of the Ministry of Health of the Russian Federation, Obninsk, Russia; Institute of Cytology and Genetics of the Siberian Branch of the Russian Academy of Sciences, Novosibirsk, Russia; Institute of Cytology and Genetics of the Siberian Branch of the Russian Academy of Sciences, Novosibirsk, Russia; Institute of Cytology and Genetics of the Siberian Branch of the Russian Academy of Sciences, Novosibirsk, Russia; Institute of Cytology and Genetics of the Siberian Branch of the Russian Academy of Sciences, Novosibirsk, Russia; Institute of Cytology and Genetics of the Siberian Branch of the Russian Academy of Sciences, Novosibirsk, Russia; State Scientific Centre of Virology and Biotechnology “Vector” of Rospotrebnadzor, Koltsovo, Novosibirsk region, Russia; National Medical Research Radiological Centre of the Ministry of Health of the Russian Federation, Obninsk, Russia; Novosibirsk State University, Novosibirsk, Russia City Clinical Hospital No. 1, Novosibirsk, Russia; State Regional Novosibirsk Clinical Tuberculosis Hospital, Novosibirsk, Russia; Research Institute of Fundamental and Clinical Immunology, Novosibirsk, Russia; Research Institute of Fundamental and Clinical Immunology, Novosibirsk, Russia; Research Institute of Fundamental and Clinical Immunology, Novosibirsk, Russia; Institute of Cytology and Genetics of the Siberian Branch of the Russian Academy of Sciences, Novosibirsk, Russia; Clinical Hospital “NeuroVita”, Moscow, Russia; Institute of Cytology and Genetics of the Siberian Branch of the Russian Academy of Sciences, Novosibirsk, Russia

**Keywords:** clonal hematopoiesis, neuroendocrine tumor, genome reconstruction technology, whole-genome sequencing, hematopoietic stem cells, клональный гемопоэз, нейроэндокринная опухоль, технология реконструирования генома, полногеномное секвенирование, гемопоэтические стволовые клетки

## Abstract

The final part of our series of studies reports the results of a pilot study of a clinical case (patient K.), indicating that therapy enabling reconstruction of the genome of hematopoietic stem cells (HSCs) reduces the frequency of SNPs in exons of the genes associated with clonal hematopoiesis of uncertain potential in a patient with a neuroendocrine tumor of the small intestine in the terminal stage of progression. The fundamental principles of the therapy were as follows. Control samples of peripheral blood mononuclear cells (baseline) were collected from the patient. CD34+ HSCs were then mobilized and collected. The HSC genome was modified using a technology enabling ex vivo correction of the nucleotide sequences of DNA chromosomes of poorly differentiated hematopoietic progenitor cells. For this purpose, the collected leukocyte suspension enriched with CD34+ HSCs was treated with fragmented deproteinized genomic DNA obtained from approximately 100 young healthy women in labor (hDNAgr). The patient was reinfused intravenously with the treated cells. Five days before reinfusion, the patient received immunosuppressive therapy. Samples of peripheral blood mononuclear cells were also collected 4, 8, 12, and 27 months after the therapeutic intervention. Full-exome sequencing of DNA isolated from selected cell samples was performed. A group of commonly accepted clonal hematopoiesis genes was selected as a criterion demonstrating changes in the genome. The following multiplex panels were used at the initial time point as well as time points after 4, 8, and 12 months: MGIEasy Exome Capture V5 Probe set, Roche KAPA HyperExome, Nanodigmbio NEXome Plus Panel v1.0, covering the entire exome and non-coding regions of the analyzed genes adjacent to exons with ~100× coverage. A ~1000× coated panel (Nanodigmbio NanOnco Plus Panel v3.0), including the genes with mutant alleles identified at the initial time point, was used to verify the identified mutations and more accurately determine their proportions in readings at the last stage of the analysis (27 months after the therapy). Whole-exome sequencing of hDNAgr (Roche KAPA HyperExome), which is used to reconstruct the genome of HSCs whose fragments collectively constitute the complete human genome, was also performed. Although the analysis of readings of whole-exome sequencing of DNA isolated from blood samples after treatment did not reveal any currently known signs of clonal hematopoiesis with uncertain potential in the patient K., we found a significant, within the framework of the selected criterion, decrease in the frequency range of SNPs to 18 % by 27 months of follow-up in 15 % cases of the heterozygous alleles (DNMT1, SF3B1(1–2)). Subsequent analysis of the frequency of occurrence of controlled SNPs and the corresponding data on the depth of their sequencing, performed using the All-FIT algorithm, indicates that by the 27th month of follow-up, the proportion of cells carrying the basic set of SNPs significantly decreases by 12 % (confidence interval (CI) = 5–16 %). In other words, in a mixture of cells heterozygous for this SNP, a population of cells carrying a homozygote without an SNP appears. Our findings may indicate that a significant proportion of cells underwent correction of the SNP to an alternative allele, and this correction was associated with the treatment of the original HSCs with hDNAgr. The detected changes in the group of clonal hematopoiesis genes suggest the possibility of correcting other loci throughout the HSCs genome. Therefore, the clinical manifestations in the development of the disease show a pronounced positive trend, which has persisted for four years, until present. It is assumed that the positive clinical outcome after therapy with reconstructed hematopoietic stem cells is associated with an increase in the regenerative potential of HSCs, which resulted from the genetic correction of unfavorable mutations in the HSCs genome.

## Abstract (ru)

В последней части цикла работ приводятся результаты пилотного исследования клинического случая (пациент К.), свидетельствующие о снижении частоты встречаемости SNP в экзонах генов, ассоциированных с клональным гемопоэзом неопределенного потенциала у пациента с нейроэндокринной опухолью тонкой кишки в терминальной стадии прогрессии при применении терапии, позволяющей реконструировать геном гемопоэтических стволовых клеток (ГСК). Основные принципы терапии состояли в следующем. У пациента отбирали контрольные образцы мононуклеаров периферической крови (нулевая точка). Затем проводились мобилизация и сбор CD34+ ГСК. Осуществляли модификацию генома ГСК с использованием технологии, позволяющей провести ex vivo коррекцию нуклеотидных последовательностей ДНК хромосом низкодифференцированных предшественников гемопоэза. Для этого собранную лейковзвесь, обогащенную CD34+ ГСК, обрабатывали фрагментированной депротеинизированной геномной ДНК, полученной от ~100 молодых здоровых рожениц (hDNAgr). Паценту внутривенно реинфузировали обработанные клетки. За 5 дней до реинфузии пациент получал иммуносупрессивную терапию. Через 4, 8, 12 и 27 мес. также забирались образцы мононуклеаров периферической крови. Было проведено полноэкзомное секвенирование ДНК, выделенной из отобранных образцов клеток. В качестве критерия, отражающего изменения в геноме, была выбрана группа общепринятых генов клонального гемопоэза. В нулевой точке, в точках через 4, 8, 12 мес. использовались мультиплексные панели: MGIEasy Exome Capture V5 Probe set, Roche KAPA HyperExome, Nanodigmbio NEXome Plus Panel v1.0, охватывающие весь экзом и некодирующие области анализируемых генов, прилегающие к экзонам с покрытием ~100×. Для верификации выявленных мутаций и более точного определения их долей в прочтениях на последнем этапе анализа, через 27 мес. после терапии, использовалась панель с покрытием ~1000× (Nanodigmbio NanOnco Plus Panel v3.0), включающая выявленные в нулевой точке гены с мутантными аллелями. Также было выполнено полноэкзомное секвенирование препарата hDNAgr (Roche KAPA HyperExome), используемого для реконструкции генома ГСК, фрагменты которого в совокупности составляют полный геном человека. Несмотря на то что анализ прочтений полноэкзомного секвенирования ДНК, выделенной из мононуклеаров крови после проведенного лечения, не позволил выявить у пациента К. известных на данный момент признаков клонального гемопоэза с неопределенным потенциалом, нами обнаружено достоверное в рамках выбранного критерия снижение диапазона частот SNP до 18 % к 27-му месяцу наблюдения в 15 % гетерозиготных аллелей (DNMT1, SF3B1(1- 2)). Последующий анализ частоты встречаемости контролируемых SNP и соответствующих данных по глубине их секвенирования, выполненный с использованием алгоритма All-FIT, свидетельствует, что к 27-му месяцу наблюдения доля клеток, несущих базовый набор SNP, значимо снижается на 12 % (доверительный интервал 5–16 %). Иными словами, в смеси клеток, гетерозиготных по этим SNP, появляется популяция клеток, несущая гомозиготу без SNP. Полученные результаты могут свидетельствовать о том, что для значимой доли клеток произошла коррекция SNP на альтернативный аллель и эта коррекция связана с обработкой исходных ГСК препаратом hDNAgr, причем обнаруженные изменения в группе генов клонального гемопоэза предполагают возможность коррекции других локусов по всему геному ГСК. Как следствие, клинические проявления в развитии патологии болезни имеют ярко выраженную положительную динамику, сохраняющуюся на протяжении четырех лет, до настоящего времени. Предполагается, что положительный клинический эффект после проведенной терапии реконструированными стволовыми клетками крови связан с усилением регенеративного потенциала ГСК, произошедшего вследствие генетической коррекции неблагоприятных мутаций в геноме ГСК.

## Introduction

Regenerative medicine is currently an actively developing medical field. Its objective is to restore the damaged or diseaseaffected tissues and organs using stem cells. Transplantation of mature stem cells is the key method employed in regenerative medicine. Two types of stem cells are used most widely: mesenchymal stem cells (MSCs) and hematopoietic stem cells (HSCs).

Mesenchymal stem cells repair damage via various mechanisms. They are activated by pro-inflammatory cytokines such as interferon gamma (IFN-γ), tumor necrosis factor alpha (TNFα), interleukin-1 beta (IL-1β), as well as by different ligands of toll-like receptors 3 (TLR3) and 4 (TLR4) (Waterman et al., 2010; English, 2013). Activated MSCs secrete a variety of cytokines and chemokines, such as fibroblast growth factor 2 (FGF2), insulin-like growth factor 1 (IGF1), transforming growth factor beta (TGFβ), prostaglandin E2 (PGE2), granulocyte-macrophage colony-stimulating factor (GM-CSF), interleukin 6 (IL-6) and interleukin 13 (IL-13), and indoleamine 2,3-dioxygenase (IDO). Through the factors secreted by them, MSCs affect many cells of the innate and adaptive immunity, including macrophages, dendritic cells, natural killer cells, T-helper and regulatory T-cells, as well as B-cells, shifting the local balance toward the anti-inflammatory signaling to promote tissue regeneration (Yin, Heit, 2021).

Due to their paracrine activity, MSCs inhibit oxidative stress and exhibit anti-apoptotic properties (Brown et al., 2019). In vitro studies have demonstrated that MSCs can migrate toward specific cytokines and chemokines, such as stromal cell-derived factor 1 (SDF-1), platelet-derived growth factor AB (PDGF-AB), IGF1, TNFα, and epidermal growth factor (EGF), which are released into the extracellular space in response to tissue injury or ischemia, and elicit anti-inflammatory responses. MSCs also release extracellular vesicles containing various growth factors promoting angiogenesis and cell proliferation, as well as microRNAs, tRNAs, and lipids, which inhibit apoptosis, facilitate cell repair, maintain the multipotency of stem cells, and contribute to angiogenesis (Maacha et al., 2020).

Another regeneration mechanism involves direct mitochondrial transfer from MSCs to damaged tissue cells via tunneling nanotubes, thus enhancing the regenerative potential of target cells and stimulating the antimicrobial activity of immune cells (Islam et al., 2012; Li X. et al., 2014; Jackson et al., 2016). MSCs are capable of differentiating into various types of connective tissue cells such as osteoblasts, chondrocytes, adipocytes, and myocytes. MSCs are found in the bone marrow, umbilical cord blood, placenta, adipose tissue, etc.; they can repair damaged cells and tissues and accelerate their regeneration (Spees et al., 2016). MSCs transplantation has shown promising potential in preclinical and clinical studies for treating numerous degenerative pathologies, including Alzheimer’s disease (Wang et al., 2018), Parkinson’s disease (Mendes Filho et al., 2018), and amyotrophic lateral sclerosis (Gugliandolo et al., 2019). Along with transdifferentiation, MSCs were shown to be able to fuse with recipient cells, including brain, muscle, intestinal, and hepatic cells, thus contributing to tissue repair in these organs (Fan et al., 2020).

The contribution of hematopoietic stem cells (HSCs) to the body’s repair potential is no less significant than that of mesenchymal stem cells. HSCs are involved in repair of tissue damage, and primarily the damage related to activity of blood cells. HSCs are capable of differentiating into all types of blood and immune cells, thus ensuring their continuous turnover in the body. There is evidence that HSCs can transdifferentiate into somatic cells, including hepatocytes, cardiomyocytes, as well as neural, epithelial, and endothelial cells, thereby contributing to regeneration of respective tissues (Lee, Hong, 2020; Li L. et al., 2021; Yuan et al., 2023). Nevertheless, there is also a viewpoint that the reparative potential of HSCs in tissue systems other than lymphohematopoietic ones is more likely to be associated with paracrine activity of HSCs rather than true transdifferentiation. It is believed that the developmental potential of HSCs, like for other adult stem cells, is limited by the innate type of stem cells and tissue (Müller et al., 2016). Similar to MSCs, HSCs actively affect cells at injury sites by secreting various molecules, including cytokines, chemokines, and growth factors, thus modulating pro- and anti-inflammatory responses and activating repair mechanisms in target cells (Schwarting et al., 2008; Li N. et al., 2010; Wright et al., 2011; Liao et al., 2018; Fast et al., 2021; Biermann, Reya, 2022).

It is known that the repair potential of hematopoietic stem cells is primarily related to their allelic diversity, underpinning normal hematopoiesis. This characteristic of HSCs is lost throughout life, and hematopoiesis in the aging organism acquires clonal features.

The classical model of hematopoiesis, positing that differentiation of HSCs into mature blood cells is a strictly hierarchical process, is evolving. The current understanding of hematopoiesis involves a concept of hematopoiesis as functioning of a heterogeneous pool of HSCs and committed progenitor cells, with numerous differentiation pathways and gradual transitions between states (Jagannathan-Bogdan, Zon, 2013; Velten et al., 2017; Watcham et al., 2019). Hematopoietic homeostasis is ensured by an intricate network of interactions between the mechanisms of transcriptional regulation, epigenetic modification, and metabolic adaptation, which are influenced by external factors, as well as humoral and local signals from the bone marrow microenvironment (Pinho, Frenette, 2019). Aging is accompanied by quantitative and qualitative changes in the hematopoietic system. The proliferative activity of hematopoietic stem cells decreases; immune activity is shifted toward pro-inflammatory responses (Weiskopf et al., 2009; Ainciburu et al., 2023). It is believed that the development of clonal hematopoiesis is one of the factors contributing to these phenotypic changes.

Normal hematopoiesis is maintained by numerous long- and short-lived hematopoietic cell clones sequentially replacing each other. The bone marrow is a tissue proliferating most rapidly in the human body. The human body produces approximately one trillion mature blood cells daily (Doulatov et al., 2012). Hematopoietic stem cells capable of differentiating into all mature blood cell lineages are underlie the hematopoietic system (Phillips, 1991; Bonnet, 2003). Clone is the progeny derived from a single hematopoietic stem cell (Siminovitch et al., 1963). The system is polyclonal upon steady-state hematopoiesis, meaning that numerous hematopoietic stem cells are simultaneously involved in maintaining normal parameters of peripheral blood cells (Drize et al., 1996; Goyal, Zandstra, 2015; Carrelha et al., 2018). Fewer than 1.3 million hematopoietic stem cells support the formation of mature peripheral blood cells throughout human life (Watson et al., 2020; Petinati, Drize, 2021). Clonal hematopoiesis is the disproportionate expansion of the progeny of several HSCs harboring specific mutations with respect to the remaining pool of clones. Clonal hematopoiesis is generally considered a biological state of the hematopoietic system rather than a disease per se.

The concept of clonal hematopoiesis has emerged after typical somatic mutations in peripheral blood cells of large patient cohorts had been detected (Genovese et al., 2014; Jaiswal et al., 2014). Mutations in a similar set of genes have been identified across all the studied groups; their prevalence was age-dependent. Over 70 different genes carrying specific mutations have been identified (Sleptsov et al., 2023). These include epigenetic regulators (DNMT3A, TET2, and ASXL1), DNA damage repair (DDR) genes (TP53, PPM1D, CHEK2, and ATM), signaling genes of cell growth (JAK2), and splicing factors (SF3B1 and SRSF2). DNMT3A, TET2, and ASXL1 were found to be the most commonly mutated genes (80–90 % of all the mutations detected) (Genovese et al., 2014; Jaiswal et al., 2014; Heyde et al., 2021; Joo et al., 2023). It is currently known that somatic mutations that have arisen and been fixed in HSCs genes, primarily those involved in regulation of epigenetic modifications and cell cycle control, are the key drivers of clonal expansion.

Somatic mutations in the genes of clonal hematopoiesis result from stochastic events; however, the accelerated pace of their accumulation is largely associated with age-related changes in HSCs, which are characterized by a decline in the overall regenerative potential (Geiger et al., 2013). Somatic mutations were found to be present in 10 % of individuals over 65 years of age vs. only 1 % in those aged under 50. The pace of mutation accumulation depends directly on the specific gene. DNMT3A-mutant clones appear at an early age and are characterized by a steady rate of emergence of new clones (approximately 5 % per year). Clones harboring mutations in splicing factor genes (SF3B1 and SRSF2) emerge at a later age; the rate of emergence of new ones is ~50 % per year. TET2-mutant clones are continuously formed and detected throughout life (Fabre et al., 2022).

The formation of mutant HSCs occurs as a result of the accumulation of mutations, and the cell loses its regenerative potential. As a result of this process, the mutated clone acquires a selective proliferative advantage, leading to clonal expansion. Clonal hematopoiesis develops, and the hematopoietic system loses the ability to repair damage in the body. Elimination of mutant HSCs clones that have accumulated somatic mutations making the cell unviable is an additional mechanism promoting clonal expansion. This defensive mechanism reduces the pool of stem and progenitor cells, thus increasing the pace of mutation accumulation through higher proliferative burden on the remaining HSCs (Sleptsov et al., 2023).

Age-related telomere shortening, caused by the semiconservative mechanism of chromosomal DNA replica- tion, increases the likelihood of DNA damage, thus also contributing to mutation accumulation (D’Adda Di Fagagna et al., 2003).

Expansion of mutant clones is not necessarily accompanied by abnormal blood parameters or clinical signs. In other words, the number of cells harboring somatic mutations in peripheral blood increases even before malignant transformation is observed. This state is known as clonal hematopoiesis of uncertain potential (CHUP) (Heuser et al., 2016; Shlush, 2018; Steensma, 2018). The key criterion for CHUP is determining the variant allele frequency (VAF) (i. e., the mutational burden, which is proportional to the number of abnormal cells). In the context of clonal hematopoiesis of uncertain potential, this value is ≥2 % (Duncavage, Tandon, 2015).

There are external factors causing somatic mutagenesis: age, genetic predisposition, pharmacotherapy, and environmental exposure risks (Joo et al., 2023). Nevertheless, the proposed variants fail to fully elucidate the molecular and cellular mechanisms of the emergence of somatic mutations in HSCs, which underlie the development of clonality in hematopoietic progenitor cells.

Based on the previously identified mechanism of extracellular DNA internalization by stem cells of different lineage, it is fair to hypothesize the following pathway for the accumulation of unfavorable SNPs in HSCs.

In peripheral tissues, mutations continuously emerge and are accumulated within cells (Shamal, 2017; Cagan et al., 2022). After apoptotic or necrotic death, cells harboring mutations release fragments containing genetic abnormalities into the bloodstream (Anker et al., 1999; Jahr et al., 2001; Laktionov et al., 2004). These fragments reach HSCs (apparently, as well as other types of stem cells) and are internalized by them via a natural mechanism due to the characteristic structure of the glycocalyx of HSCs, which is positively charged (Dolgova et al., 2014; Petrova et al., 2022; Ritter et al., 2022; Potter et al., 2024). The extracellular fragments delivered into HSCs (and potentially other stem cells) initiate terminal differentiation of HSCs, which is characterized by the emergence of pangenomic single-stranded DNA breaks (Vatolin et al., 1997; Potter et al., 2024; Ruzanova et al., 2024). This event triggers activation of the recombinational repair system of the cell and induces the so-called recombinogenic situation (Likhacheva et al., 2008). It is hypothesized that during the development of the recombinogenic situation, extracellular DNA fragments can be engaged in recombinational interactions with genomic DNA and be integrated into the genome at homologous loci by replacing native genomic sequences (Yakubov et al., 2007; García-Olmo et al., 2012). Since recombination under conditions of pangenomic single-stranded breaks is highly precise (Xu, 2015; Vriend, Krawczyk, 2017; Maizels, Davis, 2018; Zilio, Ulrich, 2021), this insertion is functional and will not disrupt the genomic sequence, except for inserting the mutation. The mutations accumulated over time can result in the development of clonal features in HSCs, with all subsequent implications. This mechanism becomes more likely in the case of activation of apoptosis or the appearance of foci of necrotic destruction in any of the body’s tissues due to pathologies that have arisen.

Therefore, analyzing the genes associated with clonal hematopoiesis has naturally become the primary focus of research aiming to elucidate the consequences of targeted therapy. If the proposed concept is valid, it will be possible to detect respective alterations in biomarker genes associated with clonal hematopoiesis in a direct experiment using treatment of HSCs with a preparation based on fragmented DNA (hDNAgr) as well as the modern whole-genome sequencing techniques. In the cases when clonal hematopoiesis of uncertain potential (CHUP) develops, the commonly accepted genes from this group, carrying driver mutations specific to this condition, can be an adequate model for detecting allele substitutions in the nucleotide sequences of these genes. If such changes are detected, the analyzed group of genes can become a marker platform indicative of ex vivo correction, which can then be extrapolated to the entire genome. However, if none of the known marker events (in the known genes) of clonal hematopoiesis are revealed, the detected changes in SNP frequencies can actually attest to alterations in the clonal composition of HSCs. In turn, this would suggest potential correction of unfavorable alleles in other genes and the emergence of clones carrying a different set of SNP alleles responsible for the regenerative potential of HSCs. In the clinical context, replacing the mutant alleles (related either to the clonal hematopoiesis genes or to other diverse genes involved in maintaining the regenerative properties of hematopoietic stem cells) with alternative favorable alleles will indicate that the original regenerative potential of HSCs has been restored.

This part of the study series reports the results of a pilot study of a clinical case, demonstrating that therapy with blood mononuclear cells enriched with CD34+ hematopoietic stem cells, which had been treated with a double-stranded human DNA preparation (hDNAgr), reduced the occurrence frequency of a certain set of SNPs marking the common genes associated with CHUP.

Under the selected criteria, we detected changes in the occurrence frequencies of certain heterozygous germline mutant alleles of the genes associated with clonal hematopoiesis: ASXL1, DNMT1, RAD21, and SF3B1(1–3). The nucleotide substitution frequency for them was reduced to 18 % of the normal distribution expected for pure heterozygous variants (50 %). It attests to the significant deviation from the expected frequency distribution for a single clonal cell lineage with the set of SNPs characteristic of the heterozygous state and the emergence of a mixture of clonal lineages harboring the investigated SNPs in different proportions.

Based on the data on allele frequencies of SNPs and the respective sequencing depth obtained using the All-FIT platform (Loh et al., 2020), we assessed the proportion of cells belonging to different clonal lineages harboring a specific SNP set and revealed statistically significant evidence for the emergence of 12 % (confidence interval (CI) ranging from 5 to 16 %) of cells carrying an alternative SNP set. Therefore, it is hypothesized that the conducted therapy led to replacement of mutant alleles with non-mutant ones. The regenerative potential of HSCs, and possibly other types of stem cells such as MSCs, was restored, which was related not only to correction in the clonal hematopoiesis genes but also involved the restoration of other mutant loci within a significant proportion of HSCs. The genetic abnormalities in these loci had caused weakening of regenerative potential. Prominent positive clinical improvement in the patient’s condition was observed.

## Materials and methods

The legal framework for conducting clinical trials. In study conduct, the authors adhered to the principles of the Declaration of Helsinki developed by the World Medical Association. The clinical part of the study was regulated by relevant treatment protocols; informed consent was obtained from the patient (Supplementary Material 1)1.

Supplementary Materials are available in the online version of the paper: https://vavilov.elpub.ru/jour/manager/files/Suppl_Oschep_Engl_30_4.zip


The hDNAgr preparation. The cells were treated using fragmented human genomic DNA (hDNAgr preparation, technical specifications TU 20.42.15-001-58179831-2023, Patent No. 2855434 dated February 2, 2026) obtained from ~100 healthy women in labor. The preparation had undergone real-time PCR verification and several assays characterizing its epidemiological purity in compliance with the regulatory documents.

Patient. Medical history. Cancer patient K., 67 years old, presenting with severe cardiovascular disorders. Medical record No. 2022/0025. The patient stayed in hospital (Clinical Hospital “NeuroVita” JSC.) from July 13, 2022 until July 29, 2022 (a total of 16 inpatient days).

Diagnosis of the underlying medical condition: C17. A neuroendocrine tumor of the small intestine (T3N0M0). The patient had undergone small bowel resection with ileostomy. The histological report dated October 29, 2018 confirmed a G2 neuroendocrine tumor with involvement of the small intestine wall, with signs of lymphovascular and perineural invasion, but without evidence of metastases. The bowel resection margins were free of tumor infiltration. Comorbidities: ischemic heart disease, post-infarction cardiosclerosis, obliterating atherosclerosis of coronary arteries.

October 29, 2018: 1 course of polychemotherapy according to the XELOX scheme. Pharmacotherapy with Octreotide Depot (until May, 2019).

October 26, 2021: disease progression, growth of foci in the liver (possibly, metastases). Pharmacotherapy with Somatuline.

November 11, 2021: chemoembolization of the segmental branches of the right hepatic artery with irinotecan. November 19, 2021: Transluminal Balloon Coronary Angioplasty (TBCA) with stenting of the right coronary artery (RCA), left anterior descending artery (LAD), and the aorta (AO).

Hypertension grade 3, risk level 4. Cardiac rhythm and conduction abnormalities: paroxysmal atrial fibrillation and paroxysmal supraventricular tachycardia. January 31, 2022: radiofrequency ablation of the slow pathway of the AV junction and the cavotricuspid isthmus. First-degree atrioventricular block. Supraventricular extrasystoles. Chronic heart failure (CHF), stage 2a, NYHA functional class 2, in the subcompensation phase

February 8, 2022: therapy with Somatuline 120 mg every 28 days. Stabilization.

Disease complications: disseminated intravascular coagulation (DIC) (May 15, 2022). Systemic inflammatory response syndrome (May 15, 2022), without pathogen identified. Liver abscess (February 16, 2022). Right lower lobe pneumonia (May 16, 2022), resolving. Oral cavity candidiasis (February 20, 2022). The patient was declared incurable by a council of oncologists; hospice care for cancer patients was recommended

Since May 11, 2022, the patient was experiencing a persistent fever (>38.0 °C). At his own discretion, the patient received Amoxiclav and NSAIDs, without any effect. He was experiencing febrile episodes (>38.0 °C) two or three times daily, which persisted until he had been hospitalized. He was admitted to the “NeuroVita” Clinic on July 13, 2022, having signs of fever of unknown origin. Complaints upon admission: right upper quadrant abdominal pain, general weakness, malaise, chest pain episodes, and arthralgia. The objective of hospitalization was to elucidate the etiology of the fever of unknown origin and to perform corrective therapy.

The patient had an advanced malignant neoplasm exhibiting signs of resistance to treatment. He met the inclusion criteria for the “Protocol of Personalized Adoptive Gene Therapy for Treating Malignant Tumors” and was enrolled in the treatment protocol based on the histologically confirmed diagnosis of malignancy; evidence for disease progression despite standard therapy or resistance to therapy; and age between 18 and 85 years.

The key phases of personalized adoptive gene therapy. All the therapeutic procedures were carried out in compliance with the protocol described in refs. (Bryukhovetskiy, Bogachev, 2023; Bryukhovetskiy, Shurdov, 2024) and the Clinical Treatment Protocol (Supplementary Material 1). At patient’s discretion and with his voluntary consent, hematopoietic blood and bone marrow cells were harvested under the program in compliance with the aforementioned protocol. The initial step involved mobilization and collection of CD34+ HSCs. Starting June 22, 2022, the patient received eight subcutaneous injections of granulocyte-colony stimulating factor (G-CSF) at 10–12-h intervals during four days following the routine hematopoietic stimulation protocol aiming to mobilize HSCs into the peripheral blood. The treatment regimen was as follows: G-CSF (figrastim) injected on days 1 and 2 at a dose of 4.4 μg/kg per day; on days 3 and 4, 8.8 μg/kg per day. On the 5th day, leukapheresis was performed, and hematopoietic stem cells from blood mononuclear cells were collected, followed by trephine biopsy and bone marrow sampling. The cellularity of the harvested material was sufficient for further manipulations. The proportion of CD34+ HSCs in the fraction of mononuclear cells (MNCs) was ~2 %. The autologous MNCs concentrate was then purified to remove red blood cells, platelets, and granulocytes using ficoll density gradient centrifugation (BioloT Ltd., Russia). The purified MNCs concentrate (leukocyte concentrate) was cryopreserved according to the standard cryopreservation procedure. Dimethyl sulfoxide (Beijing Solarbio Science &Technology Co., Ltd, China) at a final concentration of 6–10 % was added to the cell suspension. The mixture was gradually frozen to –80 °C at a rate of 1 °C/min and stored in liquid nitrogen vapor

The clinical part of the therapy. Starting July 13, 2022: immunosuppression with methylprednisolone (N2) was initiated; starting July 14, 2022: sirolimus (N4). On July 19, 2022, ex vivo gene therapy was performed via reinfusion of autologous HSCs restored using the medicinal substance hDNAgr. The sample of cryopreserved MNCs enriched with CD34+ HSCs was thawed. Immediately prior to reinfusion, the genome of the HSCs was modified using a technology enabling ex vivo correction of nucleotide sequences in chromosomal DNA of poorly differentiated hematopoietic progenitor cells. For this purpose, the collected leukocyte suspension enriched with CD34+ HSCs was washed to remove the cryoprotectant, resuspended in normal saline, and coincubated (60 min, 37 °C) with fragmented deproteinized genomic DNA (hDNAgr) obtained from ~100 young healthy women in labor. The leukocyte suspension was then washed to remove the hDNAgr. With the informed consent provided by patient K., an autologous cellbased gene therapy preparation containing 3.6×1010 autologous peripheral blood mononuclear cells in 100 mL of Sol. Polyglukini was administered intravenously via a central venous catheter under aseptic conditions at the intensive care unit. The transfusion was conducted after intramuscular premedication with Sol. Suprastini (2.0 mL). The hemodynamic parameters were monitored during the reinfusion (BP 170/70 mmHg, HR 62 bpm, SpO2 97 %). Sol. Dexametasoni 4.0 was infused intravenously after the procedure to prevent hypersensitivity reactions. Hemodynamic monitoring was performed one hour post-infusion (BP 115/60 mmHg, HR 67 bpm, SpO2 97–96 %). The patient underwent the procedure satisfactorily. No complications occurred during the procedure. Copies of the documents accompanying the “Protocol for Personalized Adoptive Immunotherapy in the Treatment of Advanced Cancer and Other Malignant Neoplasms” and the Patient Informed Consent Form are provided in Supplementary Material 1.

Patient. Follow-up of October 28, 2022. The patient feels satisfactorily and reports having hiked in the mountains and swum in a mountain lake. Contrast-enhanced CT revealed no findings indicative of cancer progression. Retroperitoneal lymph nodes were enlarged by 1–2 mm. The patient’s overall condition was interpreted as cancer stabilization.

Patient. Follow-up of April 10, 2023. On April 10, 2023, the patient was admitted to hospital for inpatient examination. His condition was satisfactory. Contrast-enhanced CT/PET imaging of the thoracic, abdominal, and pelvic organs revealed no evidence of disease progression. No abnormal tumor markers were identified. Cardiac condition had improved. Although having heart failure, ischemic heart disease, and stents in coronary arteries, the patient had experienced no chest pain over the past six months (whereas prior to therapy, he was seeking emergency care 3–4 times per week). Gout has resolved (the patient was having painful gout attacks over the past five years); hemorrhoids have become compensated. Patient’s hair color has recovered; hair has become thick and silky. The patient’s facial complexion has normalized. He is having a normal everyday life. The patient would feel absolutely healthy if he had not undergone colostomy.

Whole-genome sequencing. Cell material was collected to analyze alterations in hematopoietic stem cells after the therapy (reinfusion of mononuclear cells enriched with autologous HSCs restored using the hDNAgr preparation). Control bone marrow and peripheral blood samples were collected prior to HSCs mobilization and MNCs harvesting. Bone marrow and peripheral blood samples were also collected 4, 8, 12, and 27 months post-intervention. Whole-exome sequencing (WES) of DNA extracted from the collected samples of MNCs was performed. Furthermore, whole-exome sequencing was conducted for the hDNAgr preparation used to reconstruct the HSCs genome, whose fragments collectively represent the complete human genome sequence.

Genomic DNA was extracted from all the collected biological specimens; alterations in the genes associated with clonal hematopoiesis were analyzed by next-generation sequencing (NGS).

Targeted paired-end sequencing was conducted using an MGISEQ-G400 sequencing platform (MGI, China) utilizing multiplex panels MGIEasy Exome Capture V5 Probe Set, Roche KAPA HyperExome, Nanodigmbio NEXome Plus Panel v1.0, or Nanodigmbio NanOnco Plus Panel v3.0 (as specified in the “Test kit” column in the tables provided in Supplementary Material 2). The sequencing aimed to identify germline mutations and clonal somatic mutations in the genes associated with clonal hematopoiesis (AKT1, ASXL1, ASXL2, BCOR, BCORL1, BRCC3*, CBL, CTCF, CUX1*, DNMT1, DNMT3A, DNMT3b, ETNK1*, GNAS, GNB1*, IDH1, IDH2, JAK2, KIT, KRAS, MPL, MTOR, MYD88, NLRP3*, NRAS, PPM1D, PTEN, PTPN11, RAD21, RUNX1, SETBP1*, SF3B1, SOX2, SRSF2, TET2, TP53, U2AF1, UBA1*). The genes marked with an asterisk are not included in the Nanodigmbio NanOnco Plus Panel v3.0; in the table, the “ND” (no data) index is given for them. The targeted coverage depth is ×100 for exome panels (MGIEasy Exome Capture V5 Probe Set, Roche KAPA HyperExome, and Nanodigmbio NEXome Plus Panel v1.0) (Schema for Exome Probesets – Exome Capture Probesets and Targeted Region, n. d.), and ~×1000 or higher for the Nanodigmbio NanOnco Plus Panel v3.0 (NanOnco Plus Panel v3.0, n. d.).

Two bioinformatics pipelines were employed for analyzing the sequencing data: the first one was optimized for germline mutation detection; the second one, for somatic mutation detection.

The following software tools were used for the germline pipeline: BWA-MEM2 v2.2.1 for aligning to the human reference genome (GRCh37); StreamMD v4.3.0 for marking and removing PCR duplicates; Sambamba v1.0.1 for filtering mapped variants; GATK Picard for quality control and mapping metrics assessment; Google DeepVariant v1.6.1 for nucleotide variant identification; BCFtools v1.20 for variant filtering; and Ensembl Variant Effect Predictor (VEP) release 112 for variant annotation.

The software used for the somatic pipeline was as follows: BWA-MEM2 v2.2.1 for aligning to the human reference genome (GRCh37); StreamMD v4.3.0 for marking and removing PCR duplicates; Sambamba v1.0.1 for filtering mapped variants; GATK v4.4.0.0 for quality control and mapping metrics assessment; GATK Mutect 2 for nucleotide variant identification; FINGS v1.7.2 for variant filtering; and Ensembl Variant Effect Predictor (VEP) release 112 for variant annotation.

The coding and non-coding genetic variants are listed in individual tables in Supplementary Material 2. The coding genetic variants include those residing within the proteincoding region of the gene, including synonymous variants, as well as the variants of donor and acceptor splice sites. The variants residing outside the protein-coding regions of the gene, excluding variants of donor and acceptor splice sites, were classified as non-coding genetic variants.

The risk of pathogenicity was assessed in compliance with the ACMG (SF v2.0)/Medical and Genetic Research Center guidelines, including the following criteria: low population frequency (variant frequency in 1,000 genomes and/or ExAC C NM_015338.6 <0.01); a damaging effect (nonsense, frameshift); information about the variant is contained in locusspecific databases, etc.

The data on variant allele frequency (VAF) are reported in Supplementary Material 2 and constitute the official report on the analysis performed at the P.A. Herzen Moscow Cancer Research Institute, a branch of the National Medical Research Radiological Centre of the Ministry of Health of the Russian Federation. The data are provided in tabulated values. The data on variant allele frequencies in exons and adjacent regions listed in the summarized table are visualized using a green gradient scale.

Analysis of the frequency of occurrence of SNPs using the All-FIT software (Loh et al., 2020). The analysis was carried out using default program settings. The input data were the SNPs identified at the previous stage, along with sequencing depth data (Supplementary Material 2, Table 1). The total number of SNPs was 15. Data without removal of germline SNPs, LOH (Loss of Heterozygosity), or subclonal SNPs were included in the analysis, since the clonality set of HSCs was not supposed to contain malignant tissue variants. Only the coding SNPs for which the VAF data were available at both the initial (pre-treatment) and final (27 months posttreatment) points were used for the computation using the All-FIT software. Therefore, the sets of SNPs selected for computation did not differ, and any differences in the results were attributable exclusively to variations in frequencies, but not the set of SNPs.

Statistical analysis was conducted using the Statistica 8 software (StatSoft, Tulsa, USA). Statistical significance was assessed using the Mann–Whitney U test; the differences were considered statistically significant at Pv < 0.05.

## Results

**The summarized whole-genome sequencing data. Choosing the genome sequencing platform to analyze changes in the frequency of occurrence of SNPs**

Patient K. with a neuroendocrine tumor of the small intestine in the terminal stage of progression (stage IV, metastatic spread to the liver, lymph nodes, and the left kidney) underwent therapy with autologous MNCs enriched with HSCs treated with the hDNAgr preparation. Patient K. presenting with signs of fever of unknown origin was admitted to the “NeuroVita” clinic. The patient was declared incurable by a council of oncologists; hospice care for cancer patients was recommended. The objective of hospitalization was refining the etiology of fever of unknown origin and performing corrective therapy. The patient had an advanced malignant neoplasm exhibiting signs of resistance to treatment. During the corrective therapy, he was found to meet the inclusion criteria for the “Protocol of Personalized Adoptive Gene Therapy for Treating Advanced Cancer and other Malignant Tumors” and was enrolled in the treatment program.

After the therapy, patient K. experienced a rapid and steady improvement of his condition. Remission of the underlying disease has been observed for four years to date. It was assumed that the favorable changes in clinical manifestations of the underlying disease and comorbidities in patient K. were related to the therapeutic effect on hematopoietic stem cells present within MNCs. We have put forward a hypothesis that this impact had resulted in genetic correction of the mutant loci in hematopoietic progenitor cells (Ruzanova et al., 2024, 2025), restoring the regenerative repair potential of HSCs, which was the reason behind the prominent clinical effect.

It is known that MNCs therapy, whether administered as monotherapy or in combination with physical (e. g., ultraviolet irradiation) (Andreu et al., 1994; Greinix et al., 2000) or humoral interventions (e. g., interleukin-2) (Dummer et al., 1993; Mendelenko et al., 1997; Pal’tsev et al., 2000), or through using MNCs conditioned medium (Beer et al., 2016), elicits an anti-inflammatory response at multiple levels. This effect is attributed to factors released during MNCs apoptosis and to activation of anti-inflammatory properties in macrophages residing in various tissues (Beer et al., 2016). Furthermore, MNCs transfusion stimulates angiogenesis, which is currently extensively studied in the context of tissue tropism impairment in patients with type 1 diabetes mellitus, cardiovascular diseases (including ischemic myocardial infarction), and stroke. This effect is believed to be related to activation of endothelial progenitor cells present within the leukocyte concentrate (Zhang, Huang, 2012; Vahidy et al., 2016; Gurusamy et al., 2018; Yunir et al., 2021). However, we have failed to find any additional cases of long-lasting and diverse clinical effects of MNCs therapy aside the one observed for patient K. in the academic literature available through PubMed. As previously noted, we attribute the restoration of the regenerative potential of HSCs to correction of somatic mutations fixed in the HSCs genome. The recovered ability of HSCs to effectively repair various types of injury in different tissues and organs is additive and synergistic with respect to the regenerative potential reported for the mononuclear cell fraction, where this tissue regeneration effect may also be related to activated (but not reconstructed) HSCs, explaining the limited scope of the observable regenerative effects of MNCs therapy per se.

The fundamental elements of adoptive cell therapy (gene therapy) involve the following manipulations. As mentioned previously, the initial step includes mobilization of hematopoietic progenitor cells using granulocyte colony-stimulating factor. The therapeutic procedure entails treating MNCs with the hDNAgr preparation, exhaustive elimination of extraneous extracellular DNA from the cells, followed by reinfusion of the treated mononuclear cells into the patient’s bloodstream. The reinfused mononuclear cell fraction largely remains in its original state, except for the fact that HSCs present in the leukocyte concentrate have internalized extracellular dsDNA fragments, and processes within the cells leading to genetic reconstruction have been initiated.

The DNA fragments in the hDNAgr preparation collectively constitute a complete human genome, encompassing a broad allelic representation of healthy variants across all the genomic sequences. Following treatment, these dsDNA fragments of the hDNAgr preparation are naturally internalized by HSCs, thereby inducing the development of a recombinogenic situation (Likhacheva et al., 2008; Ruzanova et al., 2024). According to the concept of natural genome reconstruction, an exchange of homologous sequences between extrachromosomal fragments and the genomic DNA takes place via the mechanism known as single-strand assimilation (ss assimilation) (Leung et al., 1997; Langston, Symington, 2005; Symington, 2014; Yakubov et al., 2024). This exchange results in substitution of mutant alleles in the genome with alleles derived from the fragments. If the alleles within these fragments are normal, healthy variants, corrective restoration of the mutant loci will take place. These considerations suggest that the prominent clinical effect of the therapy was primarily attributable to the genetic correction of HSCs rather than to activation of undefined factors within the integral population of mononuclear blood cells.

In this study, we have put forward a hypothesis that as a result of stochastic genome reconstruction, hematopoietic stem cells have restored the regenerative potential lost throughout their life and because of the developed pathology, eventually expanding this potential to the entire body.

We conducted whole-exome sequencing (WES) of DNA extracted from the patient’s MNCs to test the hypothesis that treatment with HSCs within MNCs will be accompanied by SNP correction and the emergence of HSCs clones with nonmutant alleles, ultimately restoring the regenerative potential of HSCs and contributing to the observed clinical effects. Only the results of sequencing blood-derived MNCs samples were analyzed. Control samples were collected prior to the therapeutic intervention. MNCs samples were collected again 4, 8, 12, and 27 months post-treatment. DNA was extracted from these samples, and WES was conducted. Multiplex panels (MGIEasy Exome Capture V5 Probe Set, Roche KAPA HyperExome, and Nanodigmbio NEXome Plus Panel v1.0), targeting the entire exome with ~100× coverage (Schema for Exome Probesets – Exome Capture Probesets and Targeted Region, n. d.), were used at the initial time point and 4, 8, and 12 months post-treatment. A targeted panel with ~1,000× coverage (Nanodigmbio NanOnco Plus Panel v3.0) (NanOnco Plus Panel v3.0, n. d.), which included the genes harboring mutant alleles detected at the initial point, was employed to verify the identified mutations and more accurately quantify their proportions at the final stage of follow-up 27 months post-therapy. Non-coding regions adjacent to the exons were additionally sequenced. Furthermore, whole-exome sequencing of hDNAgr preparation, Roche KAPA HyperExome, which is used for reconstructing the HSCs genome whose fragments constitute the complete human genome, was also performed (Supplementary Material 2, Fig. 1). The utilization of multiple multiplex panels was a technical solution; their specificity for the selected coverage sufficiently aligned with the criteria required for SNP analysis (target, off-target, and duplications), making this approach adequate for assessing SNP frequencies (Schema for Exome Probesets – Exome Capture Probesets and Targeted Region, n. d.; Belova et al., 2025).

**Fig. 1.**
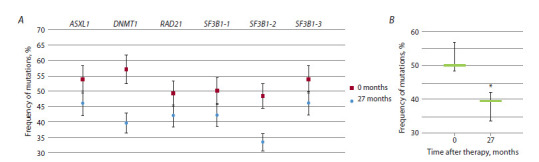
The frequency of SNPs of the analyzed genes at the final follow-up point 27 months post-intervention. A, The diagrams illustrating the overlapping of the total percentage error values for the SNP allele frequencies of three heterozygous genes at the initial and final follow-up points. B, Changes in the frequencies of SNPs in the selected heterozygous genes at the initial and final follow-up points; frequencies at the initial point fluctuate around 50 % (three SNP alleles). The median allele frequencies are compared with allowance for the total percentage error of the values obtained using different multiplex panels. * Statistically significant differences ( Pv < 0.05) as determined using the Mann–Whitney U test.

A set of genes associated with clonal hematopoiesis of uncertain potential (CHUP) was selected for analyzing genomic alterations after the treatment. This genetic platform was chosen because changes affecting the specified genes constitute the molecular foundations for the mechanism of clonal expansion of mutated HSCs clone. The resulting dominance of such clones is a key factor in the development of clonal hematopoiesis of uncertain potential compromising the body’s defense function.

It was hypothesized that dominant clones had been formed in the hematopoietic system of patient K., which could have been a confounding factor for the underlying pathology.It was assumed that the assessment of SNP frequency reduction within the selected gene group could had been one of the criteria attesting to the potential changes in the HSCs genome after treatment. The advantages of this approach are related to the ease of detecting changes in the exome by nextgeneration sequencing (NGS), the relative cost-effectiveness of the technique, and the availability of bioinformatics tools enabling targeted analysis of this specific gene set. If genetic alterations are found in the analyzed genes, it would suggest that there are high chances that similar events have occurred across the entire genome. Importantly, restoration of the regenerative potential of HSCs is more likely to be related to the recovery of other mutated loci (in which the mutations had initially weakened the regenerative potential) rather than solely to correction of mutations within the genes associated with clonal hematopoiesis. The detection of these genetic changes will be a marker of enhanced regenerative potential of the hematopoietic system and simultaneously, a marker of ex vivo correction per se. Furthermore, it is hypothesized that similar processes can occur for other types of stem cells (MSCs and endothelial progenitor cells) that are present in the leukocyte concentrate and internalize extracellular fragments (Dolgova et al., 2014), which will also be a factor significantly enhancing the overall regenerative potential of the body.

Whole-exome sequencing revealed no known driver SNPs included in the established list of genes associated with clonal hematopoiesis and responsible for its development (Genovese et al., 2014; Jaiswal et al., 2014; Heyde et al., 2021; Joo et al., 2023). It means that the patient had no systemic changes in hematopoiesis that would be detectable by genomic analysis. According to the available literature, no dominant clones of HSCs with disrupted mechanisms responsible for their regenerative potential had been formed. In other words, the clonal hematopoiesis in its classical form was not detected in this patient. Simultaneously, this fact suggests that the prominent positive changes in the patient’s condition observed after treatment are associated with genetic alterations in other mutant alleles of the HSCs genes directly involved in the aforementioned regenerative mechanisms.

Nevertheless, the analysis identified SNPs associated with the common clonal hematopoiesis genes, but these genes do not correspond to driver alleles and appear to have a germline origin (100 % occurrence frequency for a homozygote; ~50 % occurrence frequency for a heterozygote) (Supplementary Material 2, Table 1).

Primary analysis demonstrated that the frequency of some of the detected germline SNPs at the final follow-up point was significantly lower compared that at the initial point. This could mean that during the interaction between extracellular DNA fragments and hematopoietic stem cells, some SNPs affecting the regenerative potential of HSCs in the genome were replaced with an alternative allele; the hDNAgr preparation was the only possible source. In other words, a somatic correction occurred. Due to correction of mutant SNPs, a new clonal HSCs lineage could have emerged, differing from the pre-treatment one.

An important implication of this observation is that alleles of the HSCs genes harboring germline SNPs were affected. The presence of germline SNPs suggests that all the cells in the body have only this genotype, and there are no other hematopoietic stem cell populations harboring alternative alleles. This fact efficiently eliminates the questions related to the potential activation of HSCs that had existed in the quiescent state since the embryonical development, which could be triggered by the treatment. In other words, if new SNPs have appeared or existing ones have disappeared, these changes could only have occurred during the postgerminal period and resulted from somatic processes.

We needed to find approaches that would demonstrate the validity of these changes; assessment of the variant allele frequencies of the identified SNPs could be the main one.

**Analysis of the frequency of occurrence of SNPs**

Two approaches were selected to assess the frequencies of SNP occurrence: (1) comparative analysis of changes in SNP frequencies in selected genes at the initial and final follow-up points (27 months), and (2) the All-FIT algorithm (Loh et al., 2020) (Supplementary Material 2, Table 2).

Analysis of changes in the frequencies of SNP occurrence in selected genes at the initial and final follow-up points (27 months). A specific approach was used in this part of the study to prove that replacement of SNPs with alternative alleles had occurred. This approach consisted in the analysis of changes in allele frequencies within the exons of heterozygous genes. The results were initially normalized to the existing literature data, which incorporated findings obtained in different laboratories, at different time points, and employing different NGS platforms. The average error in estimating the distribution of allele frequencies within exons and adjacent non-coding regions of heterozygous genes was determined at the second stage, based solely on the findings of the present study (the “internal” experimental error). These analyses employed four different NGS platforms (Supplementary Material 2, Table 3).

Six genes (ASXL1, RAD21, DNMT1, SF3B1-1, SF3B1-2, and SF3B1-3) were selected for the comparative analysis; the frequency of SNP occurrence in these genes was ~40–60 %, suggesting that these alleles were heterozygous. The occurrence frequencies of homozygous SNPs were close to 100 % at all the analysis points. The comparison revealed a statistically significant reduction in the frequency of SNPs in three of the selected genes (ASXL1, DNMT1, and SF3B1-2) at the final follow-up point according to the selected criterion (Fig. 1). The logic and the main steps of the analysis are outlined in Supplementary Material 3. It means that the observed reduction in SNP frequency is probably associated with genetic correction of SNP that had resulted from homologous exchange of mutant genomic sequence with a non-mutant allele present in extracellular DNA fragments within the hDNAgr preparation.

The All-FIT (allele-frequency-based imputation of tumor purity) algorithm. The second approach employed the All-FIT bioinformatics platform. According to the algorithm proposed by the authors, this method allows one to determine the proportion (the “purity” according to ref. (Loh et al., 2020)) of cancer cells and accompanying somatic tissue (cells) in the resected tumor sample with a respective confidence interval (CI) using the weighted least squares method through iterative steps based on variant allele frequencies (VAF) of SNPs detected in target clinical high-throughput sequencing data and the sequencing depth for each selected allele. The simulated and clinical data were used to demonstrate the accuracy and improved performance of the All-FIT approach compared to other leading computational methods. The program was validated for specific clinical samples and showed high predictive effectiveness (Loh et al., 2020).

Following the core concept of the platform, the proposed algorithm can be used to determine the ratio between the proportion of cells harboring different SNP sets by analyzing changes in variant allele frequencies of a limited number of SNPs available for analysis as well as the sequencing depth, which shows the reliability of frequency estimates. The reduced frequency of the analyzed alleles compared to the marker (anchor) frequencies ascertained by the algorithm would attest to a decline in the number of cells carrying the generalized set of selected SNPs, being fully consistent with the objectives of our study.

The study analyzed the frequencies of SNPs in the genes associated with clonal hematopoiesis based on the initial hypothesis regarding the presence of CHUP. The findings demonstrated that the patient had no clonal hematopoiesis. However, the limited number of SNPs available for VAF analysis (together with sequencing depth data), related only to the genes of this group, allows one to conduct All-FIT analysis, which will reliably reveal any significant change in the occurrence frequency of controlled SNPs if it is detected.

**Fig. 2.**
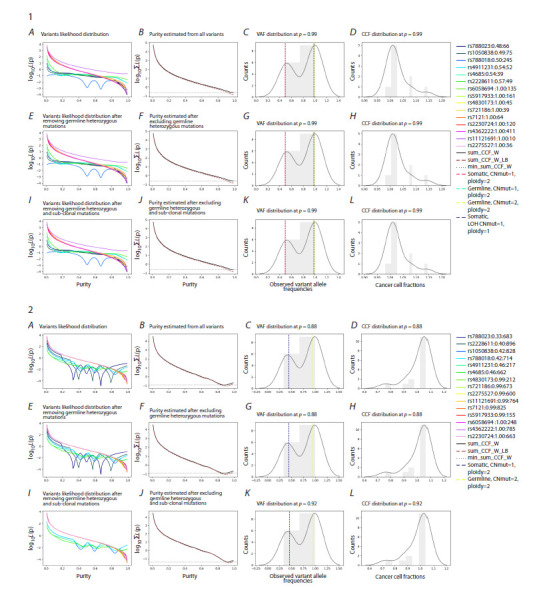
Presentation of the results of All-FIT analysis for the detected clonal hematopoiesis gene variants (Loh et al., 2020). An analysis focused on the allele frequencies of SNPs within the exons of the clonal hematopoiesis genes evaluated at two time points: at the initial (point 1) and the final follow-up points (2) (27 months post-therapy). A, The contribution of each variant to the sum of probabilities. B, The sum of probabilities across all the variants and the 2σ curve (the brown dashed line). The points of intersection between the 2σ curve and the tangent to the log of the sum of probabilities at its minimum (the gray dashed line) define the confidence interval (CI) around the calculated purity parameter p. C, The distribution of observed allele frequencies. The dashed lines are the expected allele frequencies in the included mutation models for the hypothesized p. D, The distribution of the proportion of clones (cancer cell fraction, CCF) for the hypothesized p. E–H, The results after unfiltered heterozygous germline variants had been excluded from analysis. I–L, The results after excluding the subclonal somatic variants. The legends to the figures show the mutated genes and the VAF of each detected variant.

HSCs within peripheral blood mononuclear cells were treated with the hDNAgr preparation containing a pool of ~100 different DNA samples encompassing all the sequences in the human genome (100 ×). According to the concept of natural genome reconstruction and the results of its experimental validation, DNA fragments internalized by HSCs undergo stochastic and homologous recombination with the accessible homology regions of genomic DNA. An assumption was made that a detected reduction in frequency of identified SNPs will imply that some SNPs, including those outside the analyzed SNP set, had been replaced with alternative alleles via the aforementioned homologous recombination. This genetic correction has altered the ratio between VAFs of the analyzed SNPs, which can potentially be detected by computations using the All-FIT algorithm. Extracellular dsDNA fragments internalized by HSCs within the MNCs are the best-substantiated source of this correction. Figure 2 shows the results of the comparison

The analysis revealed that the purity (the proportion of cells carrying the initial SNP set) at the initial point was 0.99 (CI, 92–99 %), which actually means that there is a single “clone”. After 27-month follow-up, the purity decreased to 0.88 (CI, 84–95 %). It means that ~12 % of cells having a different set of SNP alleles (CI, 5–16 %) have emerged. A homozygous SNP-free clonal cell lineage appears in the pool of heterozygous cells with analyzed SNPs. The most plausible explanation to these values is that some SNP alleles have been replaced with alternative alleles present in the hDNAgr preparation, which have become fixed in some HSCs lineages and are characterized by an enhanced regenerative potential.

The data obtained using the two independent approaches reinforce the conclusion that the proposed mechanism of interaction between extracellular dsDNA fragments and chromosomal DNA does actually exist. In other words, the hypothesized concept of natural genome reconstruction is viable.

The hypothetical scenario of events taking place during reinfusion of reconstructed HSCs to patient K. is provided in Supplementary Material 4.

## Discussion

Overall, our findings are consistent with the proposed concept of genome reconstruction via a natural mechanism (Ruzanova et al., 2024, 2025; Yakubov et al., 2024). The key questions of the entire study address the feasibility of non-homologous integration into the genome and the accuracy of homologous integration. The clinical applicability of the concept depends on the answer to this question.

Undoubtedly, certain events take place within the nucleus, leading to potential recombinational repair interactions between the extrachromosomal fragments of extracellular dsDNA and chromosomal DNA. It is supported by the data reported below.

We have previously demonstrated that telomeric DNA content increases upon internalization of dsDNA fragments by HSCs, which can be associated with both true integration of telomeric repeats into the genome and with alternative lengthening of telomeres (Ruzanova et al., 2025). Furthermore, circular DNA is formed, consisting of extrachromosomal extracellular fragments looping into circles and giving rise to multiple associates with chromosomal DNA strands, which persist until the metaphase and can be detected by FISH or by direct analysis of the distribution of fluorescent-labeled DNA probes (Ruzanova et al., 2025).The results of this study suggest that mutant alleles of the genes of hematopoietic stem cells (HSCs) are apparently restored, which is interpreted as projection of the core idea of the concept: stochastic substitution of various chromosomal DNA segments with homologous sequences derived from extrachromosomal fragments internalized by HSCs. An important factor in this context is that according to the findings of whole-genome sequencing conducted in (Oshikhmina et al., 2026), there is no evidence for non-homologous probe integration, indicating that homologous allele replacement is more likely to occur.Vital tests demonstrate that syngeneic reinfusion of mouse and rat HSCs treated with heterologous human DNA (hDNAgr) causes neither immediate nor long-term (one year) pathological sequelae. They also suggest that there is no illegitimate integration of foreign extrachromosomal DNA into active genomic loci; therefore, there are no genetic abnormalities manifesting themselves as an immediate functional response (Ruzanova et al., 2025).

The most important result of our study is the reported clinical case involving patient K. Long-lasting stabilization of cancer progression was achieved, accompanied by positive clinical manifestations. Along with stabilization of disease progression, there were explicit favorable effects on the cardiovascular system. The phenomenon of new hair growth demonstrates that stem cells migrated to hair follicles and were fixed in them. However, the key aspect of this clinical case is that genetic alterations in the mutant heterozygous germline alleles have been detected. In other words, our study revealed the phenomenon of somatic correction in hematopoietic stem cells, which occurred naturally through internalization of extracellular dsDNA fragments by hematopoietic progenitor cells.

The primary critical question concerning the entire technology, including the reported clinical case, is whether extracellular DNA fragments that had originally been internalized by HSCs can be preserved in their progeny for a long time (up to 4, 9, 12, and 27 months) and whether this DNA (SNPs) can be detected in leukocyte concentrate cells by whole-exome sequencing after time passes. We believe that this outcome (namely, long-term preservation of extrachromosomal extracellular DNA in the progeny of hematopoietic cells), which may give rise to the corresponding artifact, is highly unlikely. Our findings demonstrate that activated HSCs treated with dsDNA preparation continuously proliferate, at least during 15-day follow-up (Ruzanova et al., 2024). After eight months, blood cells potentially containing extracellular fragments inherited from the maternal HSCs, which is capable of internalizing up to 1 % of the haploid genome (Dolgova et al., 2013, 2016; Potter et al., 2024), will either be eliminated based on their lifespan or will constitute an extremely small proportion of the blood cell population, so that their detection will be infeasible from a quantitative perspective. Furthermore, our unpublished data indicates that extracellular DNA material is no longer detectable within cells ~22 days post-treatment. Therefore, the results of whole-exome sequencing of mononuclear cell samples derived from patient K. 4, 8, 12, and 27 months after the therapy reflect the real situation with genomic alterations and the emergence of genetically reconstructed renewed hematopoietic stem cell clones.

An interesting observation was that homozygous germline alleles of the genes have not been corrected, suggesting they are under selective pressure. Heterozygous alleles not being under selective pressure have undergone correction; the regenerative potential of the therapy has statistically significantly increased. The emergence of extracellular DNA fragments within the intracellular space of HSCs is accompanied by homologous correction and simultaneously initiates cell division (Potter et al., 2024; Ruzanova et al., 2024). The progenitor cells divide both symmetrically and asymmetrically (Potter et al., 2024; Ruzanova et al., 2024). Their progeny, resulting from both symmetric and asymmetric division, circulate in peripheral blood. It is fair to hypothesize that the genes responsible for tissue regeneration have undergone correction, thus restoring the original regenerative potential of the HSCs, and that these cells, which have acquired a new status, have caused the extensive systemic regeneration in patient K.

## Conclusion

Overall, the technology of natural genome reconstruction using the therapeutic hDNAgr (Patent No. 2855434 dated February 2, 2026) can be employed for treating patients with diseases of civilization and elderly patients without evident pathologies. Nonetheless, serious experimental studies of all subtleties of the molecular and cellular processes triggered upon interaction between a stem cell (and HSC in particular) and extracellular DNA fragments need to be conducted.

## Conflict of interest

The authors declare no conflict of interest.

## References

Ainciburu M., Ezponda T., Berastegui N., Alfonso-Pierola A., Vilas-
Zornoza A., Martin-Uriz P.S., Alignani D., … Diez-Campelo M.,
Valcarcel D., Hernaez M., Romero J.P., Prosper F. Uncovering perturbations
in human hematopoiesis associated with healthy aging
and myeloid malignancies at single-cell resolution. eLife. 2023;12:
e79363. doi 10.7554/eLife.79363

Andreu G., Leon A., Heshmati F., Tod M., Menkes C.J., Baudelot J.,
Laroche L. Extracorporeal photochemotherapy: evaluation of two
techniques and use in connective tissue disorders. Transfus Sci.
1994;15(4):443-454. doi 10.1016/0955-3886(94)90178-3

Anker P., Mulcahy H., Chen X.Q., Stroun M. Detection of circulating
tumour DNA in the blood (plasma/serum) of cancer patients. Cancer
Metastasis Rev. 1999;18(1):65-73. doi 10.1023/A:1006260319913

Beer L., Mildner M., Gyöngyösi M., Ankersmit H.J. Peripheral blood
mononuclear cell secretome for tissue repair. Apoptosis. 2016;
21(12):1336-1353. doi 10.1007/S10495-016-1292-8

Belova V., Vasiliadis I., Repinskaia Z., Samitova A., Shmitko A., Ponikarovskaya
N., Suchalko O., Cheranev V., Shatalov P., Shegai P.,
Kaprin A, Rebrikov D., Korostin D. Comparative evaluation of four
exome enrichment solutions in 2024: Agilent, Roche, Vazyme and
Nanodigmbio. BMC Genomics. 2025;26(1):76. doi 10.1186/S12864-
024-11196-Z

Biermann M., Reya T. Hematopoietic stem cells and regeneration.
Cold Spring Harb Perspect Biol. 2022;14(8):a040774. doi 10.1101/
CSHPERSPECT.A040774

Bonnet D. Biology of human bone marrow stem cells. Clin Exp Med.
2003;3(3):140-149. doi 10.1007/S10238-003-0017-9

Brown C., McKee C., Bakshi S., Walker K., Hakman E., Halassy S.,
Svinarich D., Dodds R., Govind C.K., Chaudhry G.R. Mesenchymal
stem cells: cell therapy and regeneration potential. J Tissue Eng Regen
Med. 2019;13(9):1738-1755. doi 10.1002/TERM.2914

Bryukhovetskiy A.S., Bogachev S.S. At the front edge of the fight
against clonal hematopoiesis in diseases of civilization: from bone
marrow transplantation to personalized gene-oriented and proteome-
based restitution of bone marrow and hemapoietic stem cells.
National
Association of Scientists. 2023;97(2):26-45. doi 10.31618/
nas.2413-5291.2023.2.97.845 (in Russian)

Bryukhovetskiy A.S., Shurdov M.A. Aging and Anti-aging: Biomedical
Approaches to Increasing Life Expectancy and Active Longevity.
Ridero, 2024 (in Russian)

Cagan A., Baez-Ortega A., Brzozowska N., Abascal F., Coorens T.H.H.,
Sanders M.A., Lawson A.R.J., … Gerstung M., Campbell P.J., Murchison
E.P., Stratton M.R., Martincorena I. Somatic mutation rates
scale with lifespan across mammals. Nature. 2022;604(7906):517-
524. doi 10.1038/s41586-022-04618-z

Carrelha J., Meng Y., Kettyle L.M., Luis T.C., Norfo R., Alcolea V.,
Boukarabila H., … Lord A.M., Sanjuan-Pla A., Woll P.S., Nerlov C.,
Jacobsen S.E.W. Hierarchically related lineage-restricted fates of
multipotent haematopoietic stem cells. Nature. 2018;554(7690):
106-111. doi 10.1038/NATURE25455

D’Adda Di Fagagna F., Reaper P.M., Clay-Farrace L., Fiegler H., Carr P.,
Von Zglinicki T., Saretzki G., Carter N.P., Jackson S.P. A DNA
damage checkpoint response in telomere-initiated senescence.
Nature.
2003;426(6963):194-198. doi 10.1038/NATURE02118

Dolgova E.V., Efremov Y.R., Orishchenko K.E., Andrushkevich O.M.,
Alyamkina E.A., Proskurina A.S., Bayborodin S.I., … Omigov V.V.,
Minkevich A.M., Rogachev V.A., Bogachev S.S., Shurdov M.A. Delivery
and processing of exogenous double-stranded DNA in mouse
CD34+ hematopoietic progenitor cells and their cell cycle changes
upon combined treatment with cyclophosphamide and doublestranded
DNA. Gene. 2013;528(2):74-83. doi 10.1016/j.gene.2013.
06.058

Dolgova E.V., Alyamkina E.A., Efremov Y.R., Nikolin V.P., Popova
N.A., Tyrinova T.V., Kozel A.V., … Mayorov V.I., Shurdov M.A.,
Ostanin A.A., Chernykh E.R., Bogachev S.S. Identification of cancer
stem cells and a strategy for their elimination. Cancer Biol Ther.
2014;15(10):1378-1394. doi 10.4161/cbt.29854

Dolgova E.V., Potter E.A., Proskurina A.S., Minkevich A.M.,
Chernych E.R., Ostanin A.A., Efremov Y.R., Bayborodin S.I., Nikolin
V.P., Popova N.A., Kolchanov N.A., Bogachev S.S. Properties
of internalization factors contributing to the uptake of extracellular
DNA into tumor-initiating stem cells of mouse Krebs-2 cell line.
Stem Cell Res Ther. 2016;7(1):76. doi 10.1186/s13287-016-0338-8

Doulatov S., Notta F., Laurenti E., Dick J.E. Hematopoiesis: a human
perspective. Cell Stem Cell. 2012;10(2):120-136. doi 10.1016/j.stem.
2012.01.006

Drize N., Keller J., Chertko J. Local clonal analysis of the hematopoietic
system shows that multiple small short-living clones maintain
life-long hematopoiesis in reconstituted mice. Blood. 1996;88(8):
2927-2938. doi 10.1182/blood.V88.8.2927.bloodjournal8882927

Dummer R., Becker J.C., Eilles C., Schafer E., Borner W., Burg G.
T cells migrate to tumour sites after extracorporeal interleukin 2
stimulation and reinfusion in a patient with metastatic melanoma.
Br J Dermatol. 1993;128(4):399-403. doi 10.1111/J.1365-2133.
1993.TB00198.X

Duncavage E.J., Tandon B. The utility of next-generation sequencing
in diagnosis and monitoring of acute myeloid leukemia and myelodysplastic
syndromes. Int J Lab Hematol. 2015;37(S1):115-121. doi
10.1111/IJLH.12361

English K. Mechanisms of mesenchymal stromal cell immunomodulation.
Immunol Cell Biol. 2013;91(1):19-26. doi 10.1038/ICB.
2012.56

Fabre M.A., de Almeida J.G., Fiorillo E., Mitchell E., Damaskou A.,
Rak J., Orrù V., … Campbell P.J., McKinney E.F., Cucca F.,
Gerstung M., Vassiliou G.S. The longitudinal dynamics and natural
history of clonal haematopoiesis. Nature. 2022;606(7913):335-342.
doi 10.1038/S41586-022-04785-Z

Fan X.L., Zhang Y., Li X., Fu Q.L. Mechanisms underlying the protective
effects of mesenchymal stem cell-based therapy. Cell Mol Life
Sci. 2020;77(14):2771-2794. doi 10.1007/S00018-020-03454-6

Fast E.M., Sporrij A., Manning M., Rocha E.L., Yang S., Zhou Y.,
Guo J., Baryawno N., Barkas N., Scadden D., Camargo F., Zon L.I.
External signals regulate continuous transcriptional states in hematopoietic
stem cells. eLife. 2021;10:e66512. doi 10.7554/eLife.66512

García-Olmo D.C., Picazo M.G., García-Olmo D. Transformation
of non-tumor host cells during tumor progression: theories and
evidence. Expert Opin Biol Ther. 2012;12(Suppl.1):S199-207. doi
10.1517/14712598.2012.681370

Geiger H., De Haan G., Florian M.C. The ageing haematopoietic stem
cell compartment. Nat Rev Immunol. 2013;13(5):376-389. doi
10.1038/NRI3433

Genovese G., Kähler A.K., Handsaker R.E., Lindberg J., Rose S.A.,
Bakhoum S.F., Chambert K., … Sullivan P.F., Sklar P., Grönberg H.,
Hultman C.M., McCarroll S.A. Clonal hematopoiesis and bloodcancer
risk inferred from blood DNA sequence. N Engl J Med.
2014;371(26):2477-2487. doi 10.1056/NEJMoa1409405

Goyal S., Zandstra P.W. Stem cells: chasing blood. Nature. 2015;
518(7540):488-490. doi 10.1038/nature14203

Greinix H., Volc-Platzer B., Kalhs P., Fischer G., Rosenmayr A., Keil F.,
Hönigsmann H., Knoble R. Extracorporeal photochemotherapy in
the treatment of severe steroid-refractory acute graft-versus-host
disease: a pilot study. Blood. 2000;96(7):2426-2431. doi 10.1182/
blood.V96.7.2426

Gugliandolo A., Bramanti P., Mazzon E. Mesenchymal stem cells: a potential
therapeutic approach for amyotrophic lateral sclerosis? Stem
Cells Int. 2019;2019:3675627. doi 10.1155/2019/3675627Gurusamy N., Alsayari A., Rajasingh S., Rajasingh J. Adult stem cells
for regenerative therapy. In: Teplow D.B. (Ed.) Progress in Molecular
Biology and Translational Science. Vol. 160. Academic Press,
2018;160:1-22. doi 10.1016/bs.pmbts.2018.07.009

Heuser M., Thol F., Ganser A. Clonal hematopoiesis of indeterminate
potential. Dtsch Arztebl Int. 2016;113(18):317-322. doi 10.3238/
arztebl.2016.0317

Heyde A., Rohde D., McAlpine C.S., Zhang S., Hoyer F.F., Gerold J.M.,
Cheek D., … Craig M., Swirski F.K., Nahrendorf M., Nowak M.A.,
Naxerova K. Increased stem cell proliferation in atherosclerosis accelerates
clonal hematopoiesis. Cell. 2021;184(5):1348-1361.e22.
doi 10.1016/j.cell.2021.01.049

Islam M.N., Das S.R., Emin M.T., Wei M., Sun L., Westphalen K., Rowlands
D.J., Quadri S.K., Bhattacharya S., Bhattacharya J. Mitochondrial
transfer from bone-marrow-derived stromal cells to pulmonary
alveoli protects against acute lung injury. Nat Med. 2012;18(5):759-
765. doi 10.1038/NM.2736

Jackson M.V., Morrison T.J., Doherty D.F., McAuley D.F., Matthay
M.A., Kissenpfennig A., O’Kane C.M., Krasnodembskaya A.D.
Mitochondrial transfer via tunneling nanotubes is an important
mechanism by which mesenchymal stem cells enhance macrophage
phagocytosis in the in vitro and in vivo models of ARDS. Stem Cells.
2016;34(8):2210-2223. doi 10.1002/stem.2372

Jagannathan-Bogdan M., Zon L.I. Hematopoiesis. Development. 2013;
140(12):2463-2467. doi 10.1242/dev.083147

Jahr S., Hentze H., Englisch S., Hardt D., Fackelmayer F.O., Hesch R.D.,
Knippers R. DNA fragments in the blood plasma of cancer patients:
quantitations and evidence for their origin from apoptotic and necrotic
cells. Cancer Res. 2001;61(4):1659-1665

Jaiswal S., Fontanillas P., Flannick J., Manning A., Grauman P.V.,
Mar B.G., Lindsley R.C., … Atzmon G., Wilson J.G., Neuberg D.,
Altshuler D., Ebert B.L. Age-related clonal hematopoiesis associated
with adverse outcomes. N Engl J Med. 2014;371(26):2488-2498. doi
10.1056/NEJMoa1408617

Joo L.J., Bradley C.C., Lin S.H., Scheet P.A., Nead K.T. Causes of
clonal hematopoiesis: a review. Curr Oncol Rep. 2023;25(3):211-
220. doi 10.1007/S11912-023-01362-Z

Laktionov P.P., Tamkovich S.N., Rykova E.Y., Bryzgunova O.E.,
Starikov A.V., Kuznetsova N.P., Sumarokov S.V., Kolomiets S.A.,
Sevostianova N.V., Vlassov V.V. Extracellular circulating nucleic
acids in human plasma in health and disease. Nucleosides Nucleotides
Nucleic Acids. 2004;23(6-7):879-883. doi 10.1081/NCN-200026035

Langston L.D., Symington L.S. Opposing roles for DNA structure-specific
proteins Rad1, Msh2, Msh3, and Sgs1 in yeast gene targeting.
EMBO J. 2005;24(12):2214-2223. doi 10.1038/sj.emboj.7600698

Lee J.Y., Hong S.H. Hematopoietic stem cells and their roles in tissue
regeneration. Int J Stem Cells. 2020;13(1):1-12. doi 10.15283/
IJSC19127

Leung W.Y., Malkova A., Haber J.E. Gene targeting by linear duplex
DNA frequently occurs by assimilation of a single strand that is subject
to preferential mismatch correction. Proc Natl Acad Sci USA.
1997;94(13):6851-6856. doi 10.1073/pnas.94.13.6851

Li L., Zhang S., Ge C., Ji L., Lv Y., Zhao C., Xu L., Zhang J., Song C.,
Chen J., Wei W., Fang Y., Yuan N., Wang J. HSCs transdifferentiate
primarily to pneumonocytes in radiation-induced lung damage
repair.
Aging (Albany NY ). 2021;13(6):8335-8354. doi 10.18632/
aging.202644

Li N., Zhang L., Li H., Fang B. Human CD34+ cells mobilized by
granulocyte colony-stimulating factor ameliorate radiation-induced
liver damage in mice. Stem Cell Res Ther. 2010;1(3):22. doi 10.1186/
SCRT22

Li X., Zhang Y., Yeung S.C., Liang Y., Liang X., Ding Y., Ip M.S.M.,
Tse H.F., Mak J.C.W., Lian Q. Mitochondrial transfer of induced
pluripotent stem cell-derived mesenchymal stem cells to airway
epithelial cells attenuates cigarette smoke-induced damage. Am J
Respir Cell Mol Biol. 2014;51(3):455-465. doi 10.1165/rcmb.2013-
0529OC

Liao F.L., Tan L., Liu H., Wang J.J., Ma X.T., Zhao B., Chen Y., Bihl J.,
Yang Y., Chen R.L. Hematopoietic stem cell-derived exosomes promote
hematopoietic differentiation of mouse embryonic stem cells
in vitro via inhibiting the miR126/Notch1 pathway. Acta Pharmacol
Sin. 2018;39(4):552-560. doi 10.1038/aps.2017.130

Likhacheva A.S., Rogachev V.A., Nikolin V.P., Popova N.A., Shilov
A.G., Sebeleva T.E., Strunkin D.N., Chernykh E.R., Gel’fgat
E.L., Bogachev S.S., Shurdov M.A. Involvement of exogenous
DNA in the molecular processes in somatic cell. Informatsionnyy
Vestnik VOGiS
= The Herald of Vavilov Society for Geneticists and
Breeders.
2008;12(3):426-473 (in Russian)

Loh J.W., Guccione C., Di Clemente F., Riedlinger G., Ganesan S.,
Khiabanian H. All-FIT: allele-frequency-based imputation of tumor
purity from high-depth sequencing data. Bioinformatics. 2020;
36(7):2173-2180. doi 10.1093/bioinformatics/btz865

Maacha S., Sidahmed H., Jacob S., Gentilcore G., Calzone R.,
Grivel J.C., Cugno C. Paracrine mechanisms of mesenchymal stromal
cells in angiogenesis. Stem Cells Int. 2020;2020:4356359. doi
10.1155/2020/4356359

Maizels N., Davis L. Initiation of homologous recombination at DNA
nicks. Nucleic Acids Res. 2018;46(14):6962-6973. doi 10.1093/nar/
gky588

Mendelenko M.M., Kravtsov S.A., Rodionov E.P. Clinical effectiveness
of immunotherapy with extracorporally activated blood mononuclear
cells for complex treatment of sepsis. Russ J Immunol. 1997;
2(3-4):191-198

Mendes Filho D., Ribeiro P.D.C., Oliveira L.F., De Paula D.R.M.,
Capuano V., De Assunção T.S.F., Da Silva V.J.D. Therapy with mesenchymal stem cells in Parkinson disease. Neurologist. 2018;23(4):
141-147. doi 10.1097/NRL.0000000000000188

Müller A.M., Huppertz S., Henschler R. Hematopoietic stem cells in regenerative
medicine: astray or on the path? Transfus Med Hemother.
2016;43(4):247-254. doi 10.1159/000447748

NanOnco Plus Panel v3.0 n.d. https://nanodigmbio.com/Solid-Tumor/
819.html

Oshikhmina S.G., Ruzanova V.S., Ritter G.S., Dolgova E.V., Kirikovich
S.S., Levites E.V., Efremov Y.R., … Ostanin A.A., Chernykh
E.R., Kolchanov N.A., Proskurina A.S., Bogachev S.S. Concept
of natural genome reconstruction. Part 4. Integration of extracellular
double-stranded DNA fragments into the genome of hematopoietic
stem cells and the formation of extrachromosomal intermediates.
Vavilovskii Zhurnal Genetiki i Selektsii = Vavilov J Genet Breed.
2026;30(2):163-180. doi 10.18699/vjgb-26-18

Pal’tsev A., Ovechkin A., Zakharova N., Rovina A., Leplina O.,
Shevela E., Ostanin A., Chernykh E. Cytokines in the treatment of
a generalized surgical infection. Anesteziol Reanimatol. 2000;2:
27-30 (in Russian)

Petinati N.A., Drize N.J. Clonal hematopoiesis and its role in the development
of hematological diseases. Gematologiya i Transfuziologiya
= Russ J Hematol Transfusiol. 2021;66(4):580-592. doi
10.35754/0234-5730-2021-66-4-580-592 (in Russian)

Petrova D.D., Dolgova E.V., Proskurina A.S., Ritter G.S., Ruzanova
V.S., Efremov Y.R., Potter E.A., Kirikovich S.S., Levites E.V.,
Taranov O.S., Ostanin A.A., Chernykh E.R., Kolchanov N.A., Bogachev
S.S. The new general biological property of stem-like tumor
cells. Part II: Surface molecules, which belongs to distinctive groups
with particular functions, form a unique pattern characteristic of a
certain type of tumor stem-like cells. Int J Mol Sci. 2022;23(24):
15800. doi 10.3390/IJMS232415800/S1

Phillips R. Hematopoietic stem cells: concepts, assays, and controversies.
Semin Immunol. 1991;3(6):337-347

Pinho S., Frenette P.S. Haematopoietic stem cell activity and interactions
with the niche. Nat Rev Mol Cell Biol. 2019;20(5):303-320. doi
10.1038/s41580-019-0103-9

Potter E.A., Dolgova E.V., Proskurina A.S., Ruzanova V.S., Efremov
Y.R., Kirikovich S.S., Oshikhmina S.G., … Grivtsova L.U.,
Kolchanov N.A., Ostanin A.A., Chernykh E.R., Bogachev S.S.
Stimulation of mouse hematopoietic stem cells by angiogenin and
DNA preparations. Braz J Med Biol Res. 2024;57:e13072. doi
10.1590/1414-431X2024e13072

Ritter G.S., Dolgova E.V., Petrova D.D., Efremov Y.R., Proskurina
A.S., Potter E.A., Ruzanova V.S., Kirikovich S.S., Levites E.V.,
Taranov O.S., Ostanin A.A., Chernykh E.R., Kolchanov N.A.,
Bogachev S.S. The new general biological property of stem-like tumor
cells. Part I. Peculiarities of the process of the double-stranded
DNA fragments internalization into stem-like tumor cells. Front
Genet. 2022;13:954395. doi 10.3389/fgene.2022.954395

Ruzanova V.S., Oshikhmina S.G., Proskurina A.S., Ritter G.S., Kirikovich
S.S., Levites E.V., Efremov Y.R., … Ostanin A.A., Chernykh E.R.,
Kolchanov N.A., Dolgova E.V., Bogachev S.S. A concept of natural
genome reconstruction. Part 2. Effect of extracellular double-stranded
DNA fragments on hematopoietic stem cells. Vavilovskii
Zhurnal Genetiki i Selektsii = Vavilov J Genet Breed. 2024;28(8):
993-1007. doi 10.18699/vjgb-24-106

Ruzanova V.S., Oshikhmina S.G., Ritter G.S., Dolgova E.V., Kirikovich
S.S., Levites E.V., Efremov Y.R., … Ostanin A.A., Chernykh
E.R., Kolchanov N.A., Proskurina A.S., Bogachev S.S. Concept
of natural genome reconstruction. Part 3. Analysis of changes
in the amount of telomeric DNA in colony cells as a new amplified
feature that arose during the processing of hematopoietic bone marrow
stem cells. Vavilovskii Zhurnal Genetiki i Selektsii
= Vavilov J
Genet Breed. 2025;29(4):479-495. doi 10.18699/vjgb-25-52

Schema for Exome Probesets – Exome Capture Probesets and Targeted
Region [www document]. URL https://genome.ucsc.edu/cgi-bin/hg
Tables?db=hg38&hgta_group=map&hgta_track=exomeProbesets
&hgta_table=KAPA_HyperExome_hg38_capture_targets&hgta_do
Schema=describe+table+schema (Accessed 5.27.25)

Schwarting S., Litwak S., Hao W., Bähr M., Weise J., Neumann H. Hematopoietic
stem cells reduce postischemic inflammation and ameliorate
ischemic brain injury. Stroke. 2008;39(10):2867-2875. doi
10.1161/STROKEAHA.108.513978

Shamal N. Somatic Mutations in Nature [www document]. 2017.
Available at: https://www.irb.basnet.by/ru/somaticheskie-mutacii-vprirode/
(in Russian)

Shlush L.I. Age-related clonal hematopoiesis. Blood. 2018;131(5):
496-504. doi 10.1182/blood-2017-07-746453

Siminovitch L., McCulloch E.A., Till J.E. The distribution of colonyforming
cells among spleen colonies. J Cell Comp Physiol. 1963;62:
327-336. doi 10.1002/jcp.1030620313

Sleptsov A.A., Nazarenko M.S., Puzyrev V.P. Common in atherogenesis
and carcinogenesis: clonal hematopoiesis. Russ J Cardiol. 2023;
28(10):57-65. doi 10.15829/1560-4071-2023-5511

Spees J.L., Lee R.H., Gregory C.A. Mechanisms of mesenchymal
stem/stromal cell function. Stem Cell Res Ther. 2016;7(1):125. doi
10.1186/S13287-016-0363-7

Steensma D.P. Clinical implications of clonal hematopoiesis. Mayo Clin
Proc. 2018;93(8):1122-1130. doi 10.1016/j.mayocp.2018.04.002

Symington L.S. End resection at double-strand breaks: mechanism and
regulation. Cold Spring Harb Perspect Biol. 2014;6(8):a016436. doi
10.1101/cshperspect.a016436

Vahidy F.S., Rahbar M.H., Zhu H., Rowan P.J., Bambhroliya A.B.,
Savitz S.I. Systematic review and meta-analysis of bone marrow-derived
mononuclear cells in animal models of ischemic stroke. Stroke.
2016;47(6):1632-1639. doi 10.1161/STROKEAHA.116.012701

Vatolin S.Y., Okhapkina E.V., Matveeva N.M., Shilov A.G., Baiborodin
S.I., Philimonenko V.V., Zhdanova N.S., Serov O.L. Scheduled
perturbation in DNA during in vitro differentiation of mouse embryo-
derived cells. Mol Reprod Dev. 1997;47(1):1-10. doi 10.1002/
(SICI)1098-2795(199705)47:1<1::AID-MRD1>3.0.CO;2-R

Velten L., Haas S.F., Raffel S., Blaszkiewicz S., Islam S., Hennig B.P.,
Hirche C., … Ho A.D., Huber W., Trumpp A., Essers M.A.G., Steinmetz
L.M. Human haematopoietic stem cell lineage commitment is a
continuous process. Nat Cell Biol. 2017;19(4):271-281. doi 10.1038/
ncb3493

Vriend L.E.M., Krawczyk P.M. Nick-initiated homologous recombination:
protecting the genome, one strand at a time. DNA Repair
(Amst). 2017;50:1-13. doi 10.1016/j.dnarep.2016.12.005

Wang X., Ma S., Yang B., Huang T., Meng N., Xu L., Xing Q., …
Zhang T., Wu J., Yang G.L., Guan F., Wang J. Resveratrol promotes
hUC-MSCs engraftment and neural repair in a mouse model
of Alzheimer’s disease. Behav Brain Res. 2018;339:297-304. doi
10.1016/j.bbr.2017.10.032

Watcham S., Kucinski I., Gottgens B. New insights into hematopoietic
differentiation landscapes from single-cell RNA sequencing. Blood.
2019;133(13):1415-1426. doi 10.1182/blood-2018-08-835355

Waterman R.S., Tomchuck S.L., Henkle S.L., Betancourt A.M. A new
mesenchymal stem cell (MSC) paradigm: polarization into a proinflammatory
MSC1 or an immunosuppressive MSC2 phenotype.
PLoS One. 2010;5(4):e10088. doi 10.1371/journal.pone.0010088

Watson C.J., Papula A.L., Poon G.Y.P., Wong W.H., Young A.L.,
Druley T.E., Fisher D.S., Blundell J.R. The evolutionary dynamics
and fitness landscape of clonal hematopoiesis. Science. 2020;
367(6485):1449-1454. doi 10.1126/science.aay9333

Weiskopf D., Weinberger B., Grubeck-Loebenstein B. The aging of the
immune system. Transpl Int. 2009;22(11):1041-1050. doi 10.1111/
j.1432-2277.2009.00927.x

Wright K.T., El Masri W., Osman A., Chowdhury J., Johnson W.E.B.
Concise review: bone marrow for the treatment of spinal cord injury:
mechanisms and clinical applications. Stem Cells. 2011;29(2):
169-178. doi 10.1002/stem.570

Xu S.Y. Sequence-specific DNA nicking endonucleases. Biomol Concepts.
2015;6(4):253-267. doi 10.1515/bmc-2015-0016

Yakubov L.A., Rogachev V.A., Likhacheva A.C., Bogachev S.S., Sebeleva
T.E., Shilov A.G., Baiborodin S.I., Petrova N.A., Mechetina
L.V., Shurdov M.A., Wickstrom E. Natural human gene correction
by small extracellular genomic DNA fragments. Cell Cycle.
2007;6(18):2293-2301. doi 10.4161/cc.6.18.4729

Yakubov L.A., Taranov O.S., Sidorov S.V., Nikonov S.D., Ostanin A.A.,
Chernykh E.R., Kolchanov N.A., Bogachev S.S. The concept of natural
genome reconstruction. Part 1. Basic provisions of the “natural
genome reconstruction” concept. Changing the genome of hematopoietic
stem cells using several natural cellular mechanisms that
are inherent in the hematopoietic cell and determine its biological
status as “the source of the body›s reparative potential”. Vavilovskii
Zhurnal Genetiki i Selektsii = Vavilov J Genet Breed. 2024;28(7):
696-705. doi 10.18699/vjgb-24-78

Yin C., Heit B. Cellular responses to the efferocytosis of apoptotic cells.
Front Immunol. 2021;12:631714. doi 10.3389/fimmu.2021.631714

Yuan N., Wei W., Ji L., Qian J., Jin Z., Liu H., Xu L., … He Y., Wang M.,
Tang L., Fang Y., Wang J. Young donor hematopoietic stem cells revitalize
aged or damaged bone marrow niche by transdifferentiating
into functional niche cells. Aging Cell. 2023;22(8):e13889. doi
10.1111/acel.13889

Yunir E., Kurniawan F., Rezaprasga E., Wijaya I.P., Suroyo I., Matondang
S., Irawan C., Soewondo P. Autologous bone-marrow vs.
peripheral blood mononuclear cells therapy for peripheral artery
disease in diabetic patients. Int J Stem Cells. 2021;14(1):21-32. doi
10.15283/ijsc20088

Zhang M., Huang B. The multi-differentiation potential of peripheral
blood mononuclear cells. Stem Cell Res Ther. 2012;3(6):48. doi
10.1186/scrt139

Zilio N., Ulrich H.D. Exploring the SSBreakome: genome-wide mapping
of DNA single-strand breaks by next-generation sequencing.
FEBS J. 2021;288(13):3948-3961. doi 10.1111/FEBS.15568

